# A prospective single-center study on CNI-free GVHD prophylaxis with everolimus plus mycophenolate mofetil in allogeneic HCT

**DOI:** 10.1007/s00277-021-04487-y

**Published:** 2021-03-23

**Authors:** Henning Schäfer, Jacqueline Blümel-Lehmann, Gabriele Ihorst, Hartmut Bertz, Ralph Wäsch, Robert Zeiser, Jürgen Finke, Reinhard Marks

**Affiliations:** 1 Department of Radiation Oncology, Medical Center, Faculty of Medicine, University of Freiburg, German Cancer Consortium (DKTK) Partner Site Freiburg, German Cancer Research Center (DKFZ), Heidelberg, Germany; 2 Department Hematology, Oncology & Stem Cell Transplantation, Faculty of Medicine and Medical Centre, University of Freiburg, Freiburg, Germany; 3 Clinical Trials Unit, Faculty of Medicine and Medical Centre, University of Freiburg, Freiburg, Germany

**Keywords:** Allogeneic transplantation, Everolimus, Mycophenolate mofetil, Graft versus host disease prophylaxis, Clinical trial

## Abstract

We report a single-center phase I/II trial exploring the combination of everolimus (EVE) and mycophenolate mofetil (MMF) as calcineurin inhibitor (CNI)-free GVHD prophylaxis for 24 patients with hematologic malignancies and indication for allogeneic HCT after a high dose or reduced-intensity ablative conditioning. The study was registered as EudraCT-2007-001892-12 and Clinicaltrials.gov as NCT00856505. All patients received PBSC grafts and no graft failure occurred. 7/24 patients (29%) developed acute grades III and IV GVHD (aGVHD), 16/19 evaluable patients (84%) developed chronic GVHD (cGVHD) of all grades, and 6/19 (31.6%) of higher grades. No severe toxicities related to study medication were observed. The median follow-up of all surviving patients is 2177 days. The 3-year OS was 45.2% (95% CI: 27.4–61.4%), and the 3-year PFS was 38.7% (95% CI: 22.0–55.1%). The cumulative incidence of relapse at 1 year and 3 year was 25% (95% CI: 12.5–50.0%), and 33.3% (95% CI: 18.9–58.7%), the cumulative incidence of NRM at 1 year and 3 years was 20.8% (95%CI: 9.6–45.5%), and 29.2% (95%CI: 15.6–54.4%), respectively. The utilization of CNI-free GVHD prophylaxis with EVE+MMF resulted in high rates of acute and chronic GVHD. Therefore, we do not recommend a CNI-free combination of mTOR inhibitor EVE with MMF as the sole GVHD prophylaxis. In subsequent studies, this combination should be modified, e.g., with further components like post-transplant cyclophosphamide (PTCy) or anti-thymocyte globulin (ATG).

**Supplementary Information:**

The online version contains supplementary material available at 10.1007/s00277-021-04487-y.

## Introduction

Allogeneic hematopoietic cell transplantation (allo-HCT) is an important curative treatment modality for hematologic malignancies. Allo-HCT's effect consists of myeloablation, reconstitution of donor hematopoiesis, and transmission of a new immune system, leading to the graft’s allo-reactivity against leukemia/lymphoma (GVL) or against the host (GVHD). The use of immunosuppressive agents is essential to improve the control of GVHD, but it can compromise the GVL effect. Especially in patients with malignancies and high risk for relapse, optimizing GvHD prevention while preserving GVL effect is a clinical need. The combination of CNI and methotrexate (MTX) has been the standard GVHD prophylactic regimen in allo-HCT for decades [1]. CNI such as cyclosporin A (CsA) and tacrolimus (TAC) suppress calcium-dependent gene transcription in T cells and act by suppressing the activation and production of interleukin 2 (IL-2). Side effects are acute and chronic nephrotoxicity, hepatic toxicities, hypertension, hypercholesterolemia, hyperglycemia, hypomagnesemia with convulsions, and microangiopathy (TMA). MMF is the prodrug of mycophenolic acid and suppresses T cell activation by inhibiting de novo purine biosynthesis. Studies of MMF with CsA to prevent acute and chronic GVHD in allo-HCT demonstrated conflicting results with data showing similar efficacy as CsA + MTX combined with reduced gastrointestinal toxicity and lower incidence of severe mucositis [2–4] and also in contrary data reporting reduced clinical efficacy [5–7]. Sirolimus (SIR) and everolimus (EVE) are immunosuppressive agents that inhibit the mammalian target of rapamycin (mTOR), an essential cell-cycle regulator in proliferating T cells. The anti-proliferative effect is not restricted to T cells but also observed in fibroblasts. In the context of HCT, SIR has succeeded in the treatment of steroid-refractory GVHD and in GVHD prevention [8–13]. A recent publication showed a significant reduction of acute GVHD with SIR + CsA + MMF [14]. In contrast, the combination of TAC + MMF without CsA leads to high rates of GVHD [15]. EVE is a derivative of SIR with high oral bioavailability and a shorter elimination half-life (22 vs. 72 h), enabling better control of the medication. There is little data to date on EVE's use to prevent or treat GVHD in the allo-HCT context [16]. A monocentric phase II study investigating EVE together with TAC exhibited promising activity but was stopped prematurely due to high rates of TMA [17].

Regulatory T cells (Treg) play an important role in controlling autoimmunity and GVHD. Murine GVHD models suggest CsA’s negative effect on Treg, in contrast to rapamycin (RAPA) or MMF [18]. This observation correlates with in vitro data revealing that expanding Tregs in the presence of RAPA is possible and suppression of human Tregs remain unaffected by MMF [19]. Furthermore, significantly fewer Tregs are detected in the blood of CNI-treated kidney transplant patients than of patients receiving RAPA [20]. Here we conducted a prospective single-center trial to investigate the role of a CNI-free GVHD prophylaxis regimen with EVE+MMF in allo-HCT patients with a high risk for relapse. Study goals were regimen’s feasibility regarding toxicity and protocol adherence, as well as its efficacy represented by GVHD rate, relapse rate, and overall survival (OS). Furthermore, we were interested in the immune reconstitution in these patients.

## Patients and methods

### Study design

The study was designed as a single-center phase I/II trial to evaluate the role of a CNI-free EVE/MMF combination as GvHD prophylaxis in patients with hematologic malignancies and standard indication for allogeneic transplantation. Primary endpoints were the incidence and severity of investigational drug-related toxicity, especially renal, mucosal, gastrointestinal toxicity, TMA, and the feasibility of an orally applied CNI-free GVHD prophylaxis regimen with EVE/ MMF. Secondary endpoints were hematopoietic engraftment on day + 30, incidence and severity of acute GVHD, incidence and severity of chronic GVHD within a year, progression-free survival (PFS) after 100 days and after a year, and OS after 100 days and after a year. Acute and chronic GVHD were diagnosed and graded using established criteria [21, 22]. Non-relapse mortality (NRM) was defined as the time from transplantation to death without preceding relapse. Progression-free survival (PFS) was defined as the time from transplantation to the first diagnosis of relapse or death. Overall survival (OS) was defined as time from transplantation to death of any cause. For patients, who did not experience the respective events of interest, we referred to the time from transplantation to the last documented follow-up as a censored observation. Between 2008 and 2009 when the study was done HLA class I typing with low resolution (2 digit) was performed and a single mismatch in the C locus was allowed. The trial had a two-stage study design (optimal design according to Simon), whereby treatment continuation was only allowed in the absence of graft failure and severe adverse events. After 24 patients, the study protocol was amended with alemtuzumab in the conditioning regimen due to high rates of severe acute and chronic GVHD rates and stopped prematurely due to low recruitment after additional 7 patients. The study was approved by the appropriate independent ethics committees and regulatory authorities and was done in accordance with good clinical practice guidelines, ethical principles of the Declaration of Helsinki, and national law and guidelines. All patients gave written informed consent. Before recruitment, the study was registered at www.clinicaltrialsregister.eu as EudraCT-2007-001892-12 and Clinicaltrials.gov as NCT00856505.

### Statistical analysis

Statistical analyses were performed using SAS 9.4 (SAS Institute Inc., Cary, NC, USA). For the endpoints as defined above, OS and PFS rates were estimated and displayed using the Kaplan-Meier method. Group comparisons were conducted with logrank tests and Cox regression models. Relapse mortality and NRM were regarded as competing events, similarly relapse and NRM. For these events, cumulative incidence rates were estimated using the Aalen Johansen estimator [23]. For the estimation of GVHD rates, death without prior GVHD was considered a competing event. Differences between cumulative incidence rates were investigated with Fine and Gray competing risks regression models [24].

### Preparative regimen

All patients were treated in single-patient rooms with positive-pressure high-efficiency particulate air (HEPA)-filtered air at least until engraftment, and routine laboratory tests were performed daily. Patients received prophylactic antiviral and antifungal therapy with acyclovir and fluconazole. Prophylaxis against *Pneumocystis jirovecii* (PjP) with trimethoprim/sulfamethoxazole was administered until 2 days before allo-HCT and restarted after stable leukocyte engraftment. All patients were monitored for cytomegalovirus (CMV) and Epstein-Barr virus (EBV) reactivation via PCR. Conditioning regimens included mainly fludarabine-based combinations (FBM, Flu/TT +/− treosulfan) as myeloablative reduced toxicity “RIC” or standard busulfan, cyclophosphamide as MAC regimens [25–28]. All patients received G-CSF-stimulated peripheral blood-derived grafts.

### Study medication

Everolimus (Certican®) and MMF (CellCept®) were administered orally from day − 3 to at least day + 100 and from day − 1 to day + 56, respectively. Starting dose of EVE was 1.5 mg bid. Dose adjustment was monitored by blood levels; the target level was 4–8 ng/ml for the first 3 months and tapering until day + 180. A fixed-dose of MMF was administered with 720 mg bid until day + 30 and tapered until day + 56. After 24 patients, this clinical trial was amended and additional T cell depletion by alemtuzumab was obligatory.

### Immune reconstitution

To assess the reconstitution of T cell immunity, blood samples were taken from patients at the indicated time points. 22/24 patients with EVE+MMF as GVHD prophylaxis could be analyzed. Samples from patients receiving a different GVHD prophylaxis regimen were used as a control. The frequency of T cell subsets was determined by appropriate antibody staining (CD4, CD8, CD3, CD45RA, CD62L, CCR7, CD25, CD127, Foxp3) and subsequent FACS analyses. CD4^+^CD25^+^CD127^−^ Tregs were identified by intracellular staining for Foxp3.

## Results

### Patient and transplant characteristics

Between March 2008 and April 2011, 24 patients were included (median age: 49 years; range: 21–65 years). 16 patients were male (67%), 8 female (33%). Underlying diagnoses were AML/MDS (*n* = 13), ALL (*n* = 2), CML/MPS (*n* = 3), NHL/CLL (*n* = 6). 17/24 received 1st HCT, 5/24 a 2nd HCT, and 1/24 a 3rd HCT, respectively. Five patients had autologous transplants before allogeneic transplants. For detailed patient characteristics, see Table 1. At the time of their 1st HCT, 2 patients were at low, 8 patients at intermediate, 6 patients at high and 1 patient at very high risk according to the disease risk index (DRI) [29]. Patients receiving their 2nd or 3rd HCT were not classified according to DRI but were considered at least as high risk (Suppl.Table 1). All patients received PBSC from related (7/24; 29%) or unrelated (17/24; 71%) donors in a dose of 7.0 × 10^6^ (median; range: 2.0–16.0 × 10^6^) CD34^+^ cells per kg body weight. 24/24 were matched at HLA class I (A, B, and HLA class II (DRB1). 7/24 had HLA-C mismatch (6 antigens and 1 allele) and 1 had a DQB1 mismatch.

**Table 1:** Detailed patient characteristics

#	Sex	Age	Disease	Donor	Response pre-HCT	Conditioning regimen	Best Response	Follow-up (days)
1	m	54	AML M4	Rel	REL1	FLU/BCNU/MEL	cCR1	d, REL, 623,
2	m	55	AML/ALL	Rel	cCR1	FLU/BCNU/MEL	cCR1	d, GVHD, 997
3	f	60	sAML	MUD	cCR1	FLU/BCNU/MEL	cCR1	d, GVHD, 140
4	f	53	AML M1	MUD	CR2	FLU/BCNU/MEL	CR2	A/W, CR,+IS, 2558+
5	f	27	CML TKI-failure	Rel	CR1	FLU/BCNU/MEL	CR1	d, GVHD/PTLD, 95
6	f	65	sAML	MMUD (HLA C)	REL1	FLU/BCNU/MEL	CR1	d, THROMB., 396
7	m	41	NHL-T	MMUD (HLA C)	REF	FLU/MEL/TT	CR1	d, OTH, 796
8	m	51	MDS RAEB-2	Rel	CR1	FLU/BCNU/MEL	CR1	d, GVHD, 1463
9	m	44	tAML NOS	Rel	cCR1	FLU/BCNU/MEL	cCR1	A/W,CR, +IS, 2438+
10	f	62	sAML	MUD	CR1	FLU/BCNU/MEL	CR1	d, HHV-6, 82
11	m	43	MDS RAEB-2	Rel	CR1	FLU/BCNU/MEL	CR1	A/W, CR, -IS, 2179+
12	m	61	AML	MUD	cCR1	FLU/BCNU/MEL	cCR1	A/W, CR, +IS, 2177+
13	m	36	AML M0	MUD	REL2	BU/CY	CR2	d, REL, 615
14	m	58	NHL-T	MMUD (HLA C)	CR1	FLU/TT	CR1	d, OTH, 26
15	m	58	MDS RAEB-2	MUD	CR1	FLU/BCNU/MEL	CR1	A/W, CR, +IS, 2128+
16	m	43	AML M1	MMUD (HLA C)	CR3	TREOS/CLOF/VP16	CR3	d, GVHD, 1028
17	m	55	CML	MMUD (HLA C)	CR1	FLU/TT	CR1	d, GVHD, 1674
18	m	39	AML M4	MMUD (DQB1)	CR1	BU/CY	CR1	d, HHV-6, 52
19	m	21	ALL	MUD	REL2	FLU/TT + TREOS	CR2	d, REL, 236
20	m	50	CLL	MMUD (HLA C)	REF	TT + TREOS/CLOF	REF	d, REL, 25
21	f	27	ALL	MUD	REL	TT + TREOS/CLOF	REL	d, REL, 8
22	f	61	CLL	MUD	CR	FLU/BCNU/MEL	CR	A/W, CR, +IS, 2052+
23	m	62	ALL	MMUD (HLA C)	cCR2	FLU/TT	cCR2	A/W, CR, +IS, 2037+
24	f	51	NHL-B	Rel	REL3	FLU/TT + TREOS	cCR3	d, REL, 233

Abbreviations: *m*, male; *f*, female; *AML*, acute myeloid leukemia; ALL, acute lymphoblastic leukemia; *NHL*, non-Hodgkin lymphoma; *MDS*, myelodysplastic syndrome; *RAEB*, refractory anemia excess of blasts; *NOS*, not other specified; *MPS*, myeloproliferative syndrome; CML, chronic myeloid leukemia; *Rel*, related; *MUD*, matched unrelated donor; *MMUD*, mismatched unrelated donor; *REL*, relapse; *REF*, refractory; *CR*, complete remission; *cCR*, continuous CR; *PR*, partial remission; *PD*, progressive disease; *FLU*, fludarabine; *MEL*, melphalane; *TT*, thiotepa; *TREOS*, threosulfane; *BU*, busulfane; *CY*, cyclophosphamide; *CLOF*, clofarabine; *MMF*, mycophenolate; *EVE*, everolimus; *d*, dead; *A/W*, alive and well; *+/−IS*, immunosuppression; *THROMB*, thrombosis

### Engraftment

All but one patient, who died early on day + 8, experienced neutrophile engraftment. The median time to engraftment was 17 days (range: 10–29). No graft failure occurred. All but two patients achieved stable platelet (PLT) engraftment exceeding 20.000/μl after a median of 20 days (range: 08–75). Five patients never reached PLT > 100.000/μl due to early death at days 8, 25, 52, 82, and 233, respectively. At day + 30, all evaluable patients (22/24) revealed complete donor chimerism in peripheral blood and bone marrow.

### Toxicity and infections

No study drug-related toxicity greater than grade 3 was observed. Mucositis was a common side effect, but its severity was limited to grades I–III only and there was no reported failure of oral intake of study medication due to impaired swallowing. Acute kidney injury (AKI) at higher grades (> 2) related to study medication was not observed. In one patient, who developed AKF due to tumor lysis under conditioning chemotherapy, no aggravation was seen after initiating EVE +MMF. No interstitial pneumonitis, TMA, or SOS was diagnosed. We identified no proven invasive fungal infection in the first year after HSCT. Fatal HHV-6 viral encephalitis was diagnosed in 2 patients shortly after adding steroids and CsA to medication due to severe GVHD of the gut and skin. Both patients died at day 52 and day 82 respectively.

CMV reactivation was seen in 4/14 (36%) patients at risk. All reactivations were clinically asymptomatic and detected by CMV-specific PCR in routine laboratory tests. If virus-load was > 1000 IU/ml. patients were treated with ganciclovir and valganciclovir, respectively. All CMV reactivations resolved and no CMV disease developed.

### Immune reconstitution

**Fig. 1:**
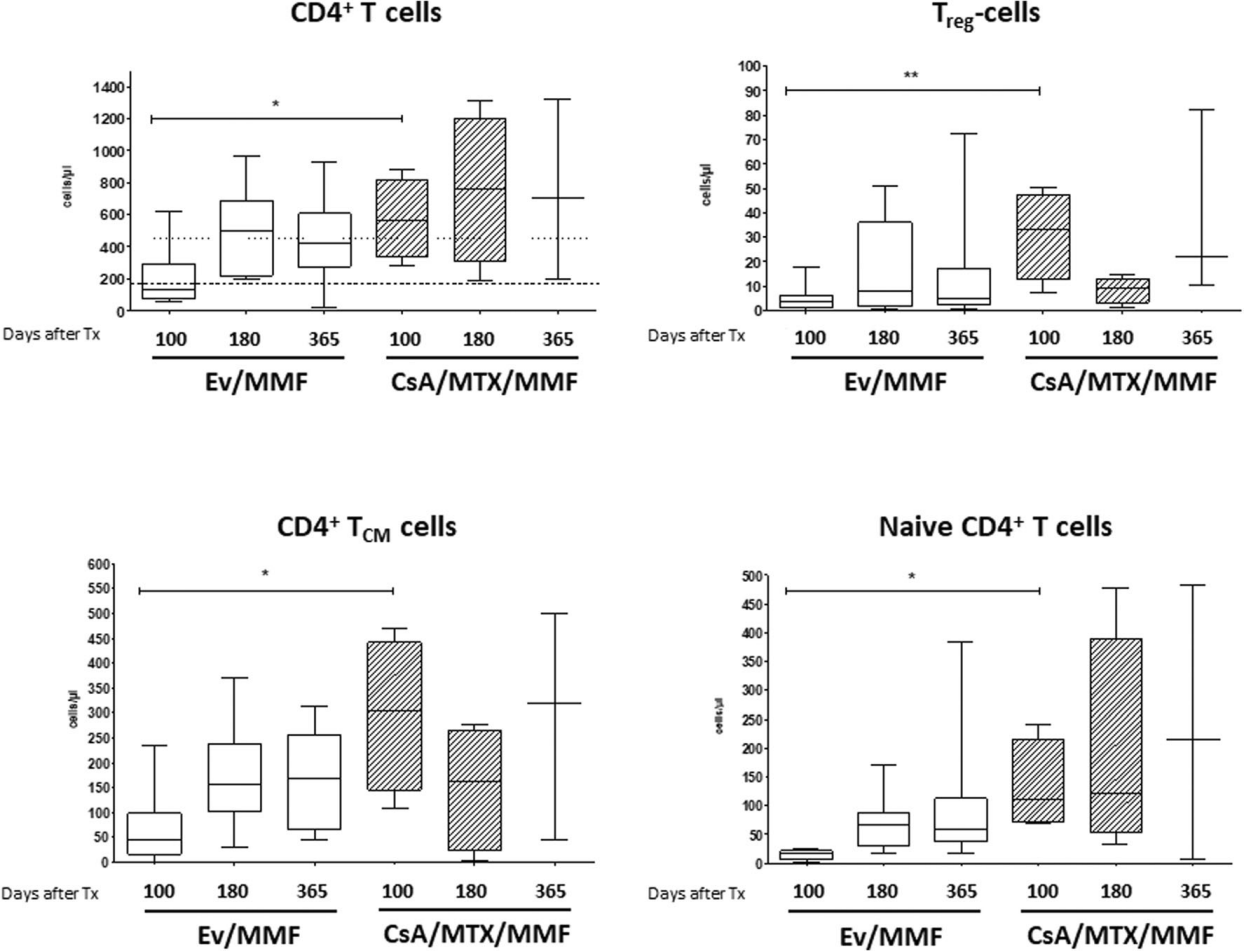
Quantitative analysis of reconstitution of CD4+ T cell subsets in patients receiving EVE+MMF or CsA+MTX/MMFas GVHD prophylaxis. Numbers indicate timepoints after HCT in days. *Statistical difference *p* < 0.05; **p < 0.005. Ev, everolimus; MMF, mycophenolate mofetil; CsA, cyclosporine; MTX, methotrexate

 Immune reconstitution analyses focused on a detailed examination of the CD4^+^ T cell compartment in consideration of the patient's GVHD prophylaxis regimen, since earlier findings in murine models suggested a better expansion of Tregs with mTOR inhibitors compared with CsA [18]. In contrast to that data, patients with EVE+MMF prophylaxis revealed significantly lower CD4^+^ T cell counts as early as day 100 after transplantation compared with historical control patients undergoing CsA-based protocols (Fig. 1). We observed this early reconstitution effect in the naive and central memory CD4^+^ T cell compartment, but most strikingly in the overall Treg cell counts. Moreover, we noted diminished overall cell counts in all three subsets up to one year after allogeneic transplantation.

### Outcome analysis

**Fig. 2:**
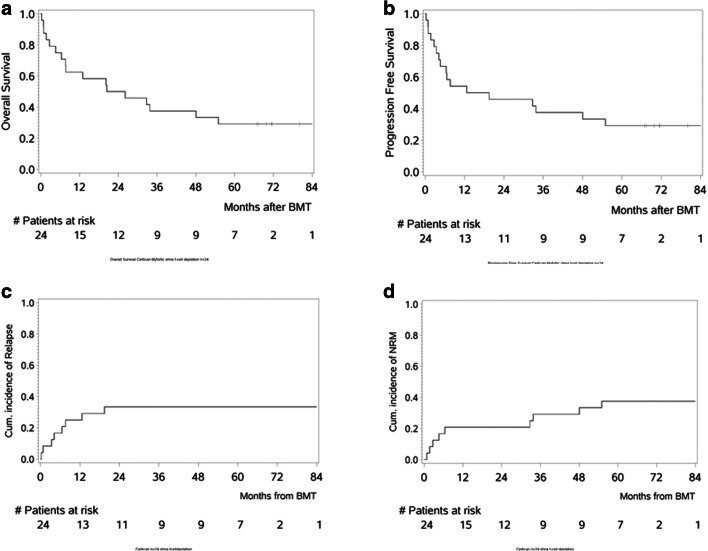
Overall survival, progression-free survival, cumulative incidence of relapse and non-relapse mortality. Cumulative incidence (%) of **a** overall survival, **b** progression-free survival, **c** incidence of relapse, and **d** non-relapse mortality in 24 patients treated with EVE + MMF for GVHD prophylaxis

The median follow-up of all surviving patients is 2177 days (range: 8–2558 days). The estimated median overall survival (OS) of the entire study population is 710 days (range: 8–2558 days). Day 100 and 1-year OS was 79.2% (95% CI: 62.9–95.4%) and 62.5% (95% CI: 43.1–81.9%), respectively (Fig. 2a). The median progression-free survival (PFS) was 489 days (range: 8–2558 days). Corresponding day 100 and 1-year PFS rates were 79.2% (95% CI: 62.9–95.4%) and 54.2% (95% CI: 34.2–74.1%), respectively (Fig. 2b). Six of 23 evaluable patients with CR after allo-HCT (one patient died due to sepsis at day + 8) suffered from relapse; 3/6 relapsing patients had AML, 2/6 NHL and 1/6 ALL. The probability of relapse at day 100 and 1-year was 8.3% (95% CI: 2.2–31.4%) and 25.0% (95% CI: 12.5–50.0%), respectively (Fig. 2c). Two relapsing patients were treated by withdrawing immunosuppression and subsequent allo-HCT. A total of 17/24 patients have died; causes were underlying malignant disease (*n* = 6), GVHD (*n* = 6), viral reactivation with HHV-6 (*n* = 2), thromboembolism (*n* = 1), and others (*n* = 2). Non-relapse mortality (NRM) was 12.5% (95% CI: 4.3–36.0%), 20.8% (95% CI: 9.6–45.4%), and 29.2% (95% CI: 15.6–54.4%) after 100 days, 1 year, and 3 years, respectively (Fig. 2d).

### GVHD

**Fig. 3:**
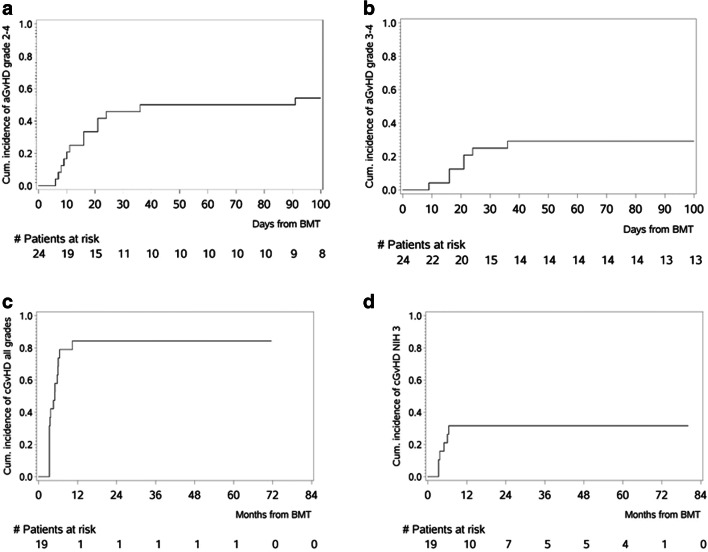
Acute and chronic GVHD. Cumulative incidence (%) of **a** aGVHD grade II-IV and **b** aGVHD grade III-IV and **c** cGVHD all grades and **d** cGVHD NIH3 with death as a competing risk in 24 patients treated with EVE + MMF for GVHD prophylaxis

Acute GVHD (aGVHD) grade II–IV occurred in 13/24 and aGVHD grade III and IV occured in 7/24 patients, respectively (Fig. 3a,b). Cumulative incidence rates at day 100 were 54.2% (grades II–IV, 95% CI: 37.5–78.3%) and 29.2% (grades III and IV, 95% CI: 15.6-54.4%) Sites affected were skin and gut, but not liver. Acute GVHD resolved mainly after steroids only, few patients needed additional therapy. Insufficient drug levels in one patient due to incompliance in an outpatient setting led to severe aGVHD refractory to all therapeutic interventions, including a subsequent allogeneic HCT.

Chronic GVHD (cGVHD) occurred in 16 of 19 evaluable patients (84.2%). Estimated cumulative incidence rates were 68.4% (95% CI: 50.4–92.9%) after 6 months and 84.2% (95% CI: 69.3–100%) after one year (Fig. 3c). Chronic GVHD severity according to NIH consensus criteria was moderate in 10 (62.5%) and severe in 6 (37.5%), respectively (Fig. 3d). Under treatment 11/16 patients (68.4%) had stable cGVHD, one patient improved and 2 complete remissions occurred. Two patients had refractory cGVHD. One patient with pre-existing obstructive lung disease (FEV1 = 52%) due to former aspergilloma resection, died in acute respiratory failure attributed to cGVHD of the lung. The second patient developed generalized severe cGVHD including skin, mouth, lung, eye and severe wasting, died from infection during cGVHD treatment.

## Discussion

The presented prospective trial is the first demonstrating a CNI-free GVHD prophylaxis with EVE+MMF in HCT for hematological patients. We observed no primary graft failure. The median time to engraftment of neutrophiles > 500/μl was 17 days without G-CSF. Delayed engraftment, as reported by other groups for combinations with RAPA and CNIs and attributed to cytotoxic effects of mTOR inhibitor EVE and MMF, was not observed; we assume that the hepatotoxic toxic effect is much more pronounced for the combination of mTOR inhibitor and CNI [30, 31]. The EVE+MMF regimen's toxicity was moderate. None of the most important adverse reactions attributable to EVE, such as interstitial pneumonitis, cutaneous reactions, or mucosal ulcers, were observed. Of note, neither we observed Transplant-associated thrombotic microangiopathy (TMA) nor sinusoidal obstruction syndrome (SOS), which is reported to occur frequently in EVE+TAC patients [17]. Nevertheless, it should be noted that our study only included 2 patients receiving a standard-dose Bu/Cy conditioning, where TMA and SOS more commonly occur.

The expected 4-year OS and cumulative risk of relapse (CRR) in patients receiving their 1st HCT with disease risk index (DRI) comparable with our cohort, is 33.2% and 45.5%, respectively [29]. In patients receiving their 2nd or 3rd HCT, the expected 2-year OS and 2-year CRR is even lower with 21–29% and 40–44%, respectively [32, 33]. The median OS of patients in our trial was 23.3 months with a corresponding 2-year OS of 50% (95% CI 30–70%) and 4-year OS 37.5% (95% CI 18.1–56.9%). CRR at 1 year and 5 years was 25% (95% CI: 12.5–50%) and 32.26% (95% CI: 12.5–50%), respectively. Regarding OS and CRR, our results are comparable with the literature.

In contrast to murine models, we documented neither earlier CD4^+^ T cell lymphocyte recovery in patients receiving EVE+MMF compared with CsA patients, nor enhancement of the numbers of CD4^+^CD25^+^ Tregs [18, 34]. Whether the use of steroids as a confounding factor caused this discrepancy cannot be excluded.

Acute GVHD of grades II–IV occurred in 54.2% of patients, which is a higher incidence rate than reported in patients receiving SIR+TAC, EVE+TAC, or SIR+MMF+ATG, but lower than in those receiving CNI+MTX [13, 26, 30, 31]. It remains unexplained, whether these higher rates are caused by different characteristics of patients treated in those studies, the previously described immune reconstitution patterns of our patients treated with EVE, or the usage of T cell depletion in at least one of the mentioned studies.

Chronic GVHD of all grades occurred in 84.2% of our patients, comparable with regimens using EVE+TAC, but higher than in patients undergoing additional T cell depletion by ATG [31, 35]. Therefore, the study protocol was amended with T cell depletion and results appear superior (data not shown), but due to low numbers (*n* = 7), no firm conclusions can be drawn.

In conclusion, our data show—to our knowledge—the first prospective trial investigating aCNI-free GVHD prophylaxis regimen composed of the mTOR inhibitor everolimus and mycophenolate mofetil in the absence of additional GVHD preventing measures. The acute toxicity was moderate and no unexpected toxicities occurred. Formerly reported microangiopathic complications in CNI/ mTOR-inhibitor combinations were not observed. The main limitation of this trial is the heterogeneity of included patients with different diseases, conditioning regimens, and risk profiles. For the purpose of this type of phase I/II trial, to test the toxicity and early efficacy of a novel GVHD prophylaxis regimen, the protocol allowed the inclusion of patients with different diagnoses/ states of disease. This approach is not unusual and the interpretation of the results is carefully done accordingly.

Due to high rates of acute and chronic GVHD, the combination of everolimus (EVE) and mycophenolate mofetil (MMF) should not be considered an acceptable GVHD prophylaxis regimen. However, regarding toxicity and relapse rate EVE and MMF with other combination partners - preferably T cell depleting agents to reduce GVHD rates - might be worth further studying.

## Supplementary information

ESM 1 (DOCX 17 kb)

## Data Availability

Data are stored digitally and as “hard copy” in clinical trial files.
