# Supplementary material for: A prospective single-center study on CNI-free GVHD prophylaxis with everolimus plus mycophenolate mofetil in allogeneic HCT
Source: Ann Hematol. 2021 Mar 23;100(8):2095–103. doi: 10.1007/s00277-021-04487-y (PMC8285343; doi:10.1007/s00277-021-04487-y)
Supplement: Supplementary file 1 — (DOCX 17 kb) [file 277_2021_4487_MOESM1_ESM.docx]

### Suppl. Table 1:

| # | Disease | Mol.Features | Disease  Risk | Stage  Risk | DRI | #allo-HCT |
| --- | --- | --- | --- | --- | --- | --- |
| 1 | AML M4 | FLT3-ITD pos., NPM1 mut, CEBPA WT | low | high | **2** | 1 |
| 2 | AML/ALL | NA | int | low | **2** | 1 |
| 3 | sAML | NA | int | low | **2** | 1 |
| 4 | AML M1 | NA | int | high | **3** | 1 |
| 5 | CML TKI-failure | 46, XX, t(9;22)(q34;q11)[3], 46,XX, der(6) t(6;17) (p21;q12), t(9;22)(q34;q11), der(17) del(17)(p11) del(17)q(119)[3] | low | high | **2** | 1 |
| 6 | sAML | complex Karyotype - e.g. der(5)t(5;17), der(7)t(7;18) | high | low | **3** | 1 |
| 7 | NHL-T | complex Karyotype | high | high | **4** | 1 |
| 8 | MDS RAEB-2 | 45,X,-Y [15], 46,XY [5] | int | low | **2** | 1 |
| 9 | tAML NOS | FLT3-ITD pos., FLT3-TKD WT, NPM1 mut, MLL-PTD neg- | high | low | **3** | 1 |
| 10 | sAML | 5q-,trisomy 21, monosomy 7 | high | low | **3** | 1 |
| 11 | MDS RAEB-2 | 46,XY, t(3;8)(q26;p23) [15/21], vor TX: 46,XY, t(3;8)(q26;p23) [12/20] | high | low | **3** | 1 |
| 12 | AML | 46,XY,del(6)(q15q27) [4/17), FISH: del 6q21, | high | low | **3** | 1 |
| 13 | AML M0 | CN | int | low | **2** | 1 |
| 14 | NHL-T | NA |  |  | **_*_** | 2 |
| 15 | MDS RAEB-2 | NA | int | low | **2** | 1 |
| 16 | AML M1 | CN |  |  | **_*_** | 3 |
| 17 | CML | bcr/abl pos. complex Karyotype: 45,XY,i(8)(q10),t(9;22)(q34;q11),-20,der(20)t(1,20)(q21;p13) | low | high | **2** | 1 |
| 18 | AML M4 | 46 XY, t(6;9),(p23;q34), FLT3-ITD pos. | low | high | **2** | 1 |
| 19 | ALL | bcr/abl neg. |  |  | **_*_** | 2 |
| 20 | CLL | del13q14, del20q12-q13.2, IgVH non mut. |  |  | **_*_** | 2 |
| 21 | ALL | bcr/abl neg. |  |  | **_*_** | 3 |
| 22 | CLL | del13q14, del15q26 | low | high | **2** | 1 |
| 23 | ALL | bcr-abl pos |  |  | **_*_** | 2 |
| 24 | NHL-B | NA |  |  | **_*_** | 2 |

**Suppl.Table 1:**

### Calculating disease Risk index (DRI) according to Armand et al.^24^ *Patients with 2nd or 3rd allo-HCT were not classified but considered as at least high-risk.
